# From each according to means, to each according to needs? Distributional effects of abolishing asset-based payments for residential care in Austria

**DOI:** 10.1186/s12939-022-01639-y

**Published:** 2022-03-19

**Authors:** Ricardo Rodrigues, Cassandra Simmons, Tamara Premrov, Christian Böhler, Kai Leichsenring

**Affiliations:** European Centre for Social Welfare Policy and Research, Vienna, Austria

**Keywords:** Access, Demand, Utilization of services, Aging, Elderly, Geriatrics, health care costs, health care financing, Insurance, Premiums, Long-term Care, Home Care, Nursing Homes

## Abstract

**Background:**

Most countries in Europe require out-of-pocket payments (OPPs) for nursing homes based on users’ income and often assets. This was also the case in Austria until 2018 when asset-based contributions to residential care —denoted the ‘Pflegeregress’ – were abolished, leaving a shortfall in revenue. We aim to determine how the Pflegeregress was distributed across different groups in Austria prior to 2018, what the distributional consequences of its abolishment were, and what the distributional impact of different financing alternatives would be.

**Methods:**

Circumventing data availability issues, we construct a micro-simulation model using a matched administrative dataset on residential care users receiving the Austrian care allowance (Pflegegeldinformation, PFIF, HVB, and Pflegedienstleistungsstatistik, Statistik Austria) and survey data (SHARE, wave 6). Using this model, we estimate the expected duration of residential care and OPPs under the Pflegeregress of a representative sample of older people aged 65 + in Austria, as well as OPPs under budgetary neutral financing alternatives to the abolished asset-based contribution, namely an inheritance tax and a social insurance scheme. The distributional impact of abolishing the Pflegeregress and these alternative scenarios is assessed through a number of measures, such as ability to pay, Concentration Indices (CI) and a needs-standardized measure.

**Results:**

We find that lower income individuals and homeowners disproportionately contributed to asset-based OPPs for residential care prior to 2018, due in large part to their higher use of residential care and the low asset-exemption thresholds. These groups were therefore the largest beneficiaries of its abolishment. The alternative financing scenarios tested would result in a more progressive distribution of payments (i.e. concentrated on more affluent individuals).

**Conclusion:**

Our findings indicate the limited ability of asset-based OPPs to target those with higher assets, thus questioning the fairness of these instruments for financing residential care facilities for older people in Austria. Findings also suggest that the parameterization of such OPPs (such as asset exemption thresholds) and patterns of residential care use are key variables for assessing the distribution of asset-based OPPs for residential care use. Policy alternatives that decouple payments from use would entail greater transfers from healthy to less healthier individuals.

**Supplementary Information:**

The online version contains supplementary material available at 10.1186/s12939-022-01639-y.

## Background

Demographic ageing has raised concerns about the fiscal sustainability and affordability of care for older people. Out-of-pocket payments (OPPs) by users and/or their families are relatively common when entering a residential care facility (e.g. nursing home or retirement home) [1]. Most countries use asset tests to determine these OPPs, meaning that residents must spend down their assets before being eligible for additional public benefits [2]. These are in practice means-tested asset-based OPPs for residential facilities, that are meant to improve allocative efficiency by curbing ‘frivolous’ consumption of costly care as residential care is, and reducing moral hazard [3]. This hinges however, on the sensitivity of use of residential care to prices. If use is insensitive to OPPs (i.e. inelastic), changes to the value of asset-based OPPs will have a limited impact on reducing frivolous consumption. Another underlying rationale for asset-based OPPs is that individuals should contribute to care costs according to their ability to pay. Asset-based OPPs have also potential distributional implications. These OPPs are borne by users of services who are more likely to be unhealthy and poor [4]. Depending on the exemption thresholds of asset-based OPPs, poorer individuals may pay a higher share of these OPPs (i.e. payments made by poorer individuals represent a higher share of the total sum of asset-based OPPs paid) and these may represent a higher percentage of poorer individuals’ ability to pay compared to richer individuals. Furthermore, if use is insensitive to the costs borne by users, changes to the generosity of public long-term care (LTC) systems would entail a recomposition of the share of total costs that are paid by taxpayers and users of care [5]. For example, in the case of an increase in generosity (e.g. by reducing the amounts of asset-based OPPs by care users or increasing exemption thresholds) a larger share of total costs, relative to before, would be financed by taxpayers rather than users, assuming that patterns of use remain equal.

According to a recent study on the rules governing asset-based OPPs, anyone with even moderate assets may expect to contribute substantially to the costs of residential care across the OECD region [2]. Against this backdrop it is perhaps unsurprising that there is an absence of popular support for asset-based OPPs [6] and that their abolishment or reform features prominently in the policy debate [7]. Due to lack of data on actual payers of residential care, there is however a dearth of comparable evidence on the distributional impact of OPPs for this type of care, with only a few existing studies focusing on England, the US and more recently the Netherlands [8–15]. According to these studies asset-based OPPs are concentrated in a relatively small share of users of residential care, although the average amounts conditional on paying asset-based OPPs are high. These same studies show that limiting or abolishing asset-based OPPs leaves home owners significantly better off, but the impact across income groups is less clear. Users from the lower and higher income quintiles seem to benefit the least from such reforms, since the former are unlikely to hold significant assets, while the latter are able to fully fund costs from their own income. This study adds to this existing body of literature by focusing on the recent abolishment of asset-based OPPs for residential facilities in Austria and analyzing the distributional implications of this policy measure.

Residential facilities, including care and nursing homes, are financed through OPPs and public subsidies in Austria. The latter mostly take the form of a universal care allowance with seven care levels based on a needs-assessment. Additional social assistance benefits are available if both pension income and care allowance are insufficient to cover costs. Until 2018, users had to pay OPPs based on their income and in addition were required to spend down their assets before being able to draw on social assistance – i.e. there was an additional means-tested asset-based OPP denominated *Pflegeregress*. Regulations varied across regions, but generally, housing and financial assets above a certain threshold were considered and there was a ‘look back’ period for transfer of assets (see Appendix 1 for details). The share of costs covered from total OPPs ranged from 41.5% in Carinthia to 58.8% in Vienna [16]. In 2018, the asset-based OPP for residential care facilities was abolished without additional taxes being raised to replace the resulting revenue shortfall. Unavailability of data on actual payers or payments from the Pflegeregress means there are limited data on how the benefits of such policy were distributed, even if lower income individuals are more likely to require residential care in Austria due to the positive gradient between health and income [17].

We aim to answer the following questions: Who contributed to the Pflegeregress and therefore benefited from its abolishment? What would be the distributional consequences of different alternative financing scenarios? To this end, we use a micro-simulation model and matched administrative and survey data to investigate how the Pflegeregress was distributed across income and house ownership groups in Austria before 2018 and the distributional consequences of its abolishment, as well as of alternative financing models. The method employed contributes to developing practical ways for conducting distributional analysis of care policies for older people in the face of severe data constraints.

## Methods and data

### Linking data for estimated duration of residential care use

In absence of data on actual payers of the Pflegeregress, we construct a micro-simulation model to estimate the expected duration of residential care and OPPs on a representative sample of community dwelling older people aged 65 + for Austria [11, 12]. We use individual level data on care allowance recipients in Austria [18], periodic life tables [19], and individual level data on care allowance recipients using residential care [20] to estimate the remaining portion of life spent in residential care using the Sullivan method [21]. The Sullivan method is a lifetable approach used to calculate the remaining years spent in diminished or full health. We adapt the method to calculate remaining life spent in residential care stratified by age, gender and care allowance level. All data refer to 2015 (see Appendix 1 for more in-depth description of the adapted Sullivan method and micro-simulation model).

We assign care levels to individuals aged 65 + in the Survey of Health, Ageing and Retirement in Europe (SHARE, wave 6) [22] for Austria (*N* = 2,221) based on Brugiavini et al. [23] Using the official list of limitations/tasks used to determine eligibility for the care allowance, we allot the prescribed time for each task that individuals in SHARE report difficulties with (see Appendix 1 for details). We then assign individuals into likely care levels based on the estimated number of hours of care required. Finally, the real-life distribution of care allowance recipients is superimposed onto the SHARE dataset by age and gender using a least-distance algorithm, taking care level as the distance-minimizing variable. Each observation of SHARE is then assigned a corresponding expected duration of residential care in the given year according to this newly matched care allowance level, gender and age. We thus simulate the distribution of individuals 65 + in residential facilities in Austria for 2015 and the average expected duration of residential care. According to this simulated distribution, women on average have a longer expected duration of residential care (7.4 months) versus men (5.8 months). By quintiles, the bottom quintile would spend the longest duration in residential facilities (7.5 months) versus the top quintile (6.1 months). Finally, disaggregated by care levels, expected duration increases with care level, peaking at individuals assigned to care level 5 with 13.6 months, before decreasing across the two highest levels (see Appendix 1, Table A4).

### Constructing the micro-simulation

To estimate OPPs, the micro-simulation model considers individual’s income, household assets, family composition, asset exemptions, monthly living allowance exemptions (i.e. income that users can retain) and costs of residential care for 2015 (see Appendix 1 for detailed rules). We operationalize income as the net income resulting from the sum of all income components in SHARE, including social benefits. Wealth is measured as household net worth, including all household financial and real assets net of any debt. Throughout, the ranking variable for distributional analysis is equivalized net income (OECD square root scale).

By simulating OPPs for a one-year time horizon, we avoid the strong assumptions underlying dynamic long-term micro-simulations used for estimating lifelong costs, which we also deem more appropriate given the nature of the administrative data used. This allowed comparing the income distribution of users of residential facilities in our simulation with administrative data on actual users in one region for which we were able to access data (Vienna) for 2011 to assess external validity. This sensitivity analysis indicates that our simulation captures the income profile of residential care users (see Appendix 2 for details).

The micro-simulation model is also able to estimate the distributional impact of financing alternatives to the Pflegeregress. Two financing alternatives based on scenarios included in previous studies [11, 12] and on the discussions surrounding the abolishment of the Pflegeregress [24] are considered: applying an earmarked inheritance tax, and introducing a social insurance contribution towards residential care. The parameters of the two alternative scenarios are as follows. Under the ‘inheritance tax’ scenario, a tax rate of 31.48% is applied to individual wealth above a threshold of €300,000 for those in the sample expected to pass away during 2015. The probability of dying was calculated using information from the periodic life table for 2015 [19], stratified by gender, age and care level. In the ‘social insurance’ scenario, a social insurance contribution (SIC) of 2.27% is applied to the income of those 65 + exceeding €405,98 per month, to a maximum income of €4,650 per month (see Appendix 1). Both scenarios are budgetary neutral in that they would completely offset the estimated cost of abolishing Pflegeregress.

### Assessing distributional impact

To assess the distributional impact of OPPs and specifically that associated with the abolishment of asset-based OPPs and alternative financing scenarios, we use four measures of distribution [25]. The first displays average OPPs per quintile in percentage of the average income for the same quintile, reflecting ability to pay for care per quintile (AP), i.e. close to the concept of catastrophic health expenditure [26]. The second metric calculates the share of cumulative OPPs by each quintile, ranging from 0 to 1, with 0.2 denoting a perfectly equal distribution of OPPs across quintiles. The third measure is given by Concentration Indices (CI) and respective Concentration Curves (CC) using equivalized household income as the ranking variable [27, 28]. The CI is a commonly used indicator to assess socio-economic inequalities in health or healthcare use and measures relative inequality in one variable (e.g. OPPs) over the distribution of another (e.g. equivalized household income). The CC provides a graphic representation of the cumulative proportion of OPPs paid against the cumulative proportion of the population ranked from the poorest to the richest. The CI is twice the area between the CC and the 45° line indicating perfect equality. The fourth measure estimates the degree of inequality that remains after controlling for differences in need [29]. We use Ordinary Least Squares (OLS) to estimate:

1 $${OPP}_{i}= \alpha +{\sum }_{k}{\beta }_{k}{N}_{ki}+ {\sum }_{j}{\upgamma }_{j}{\mathrm{Z}}_{ij}+ {\upvarepsilon }_{i}$$

Where actual OPP_i_ is regressed on a vector of need variables ${N}_{k}$ (self‐rated health, number of limitations with Activities of Daily Living (ADL) and Instrumental Activities of Daily Living (IADLs), number of chronic illnesses, mental health, cognitive impairment, age, and gender) and non-need variables ${\mathrm{Z}}_{j}$ (education, marital status, number of children, income quintile). Using the parameter estimates from (1) and sample means of the non-need variables ($\overline{{\mathrm{Z} }_{j}}$), we estimate the predicted value of OPPs: $\widehat{{OPP}_{i}}$. The needs-adjusted OPPs are obtained by:

2 $${OPP}_{i}^{need}={OPP}_{i}-\widehat{{OPP}_{i}}+\overline{OPP }$$

Where $\overline{OPP }$ is the sample average of OPPs. ${OPP}^{need}$ can then be averaged across income quintiles and compared. Alternatively, the horizontal inequity index (HI) can be used as a synthetic metric for how much OPPs paid deviate from needs [30]; an approach similar to other studies that used HI to assess the distribution of care costs [29, 31]:

3 $$HI=CI\left(OPP\right)-CI\left(\widehat{OPP}\right)=CI({OPP}^{need})$$

## Results

Table 1 shows how different types of OPPs (total, income-based and asset-based) estimated by the micro-simulation model were distributed by income quintile and home ownership status. In absolute values, total OPPs are increasing with income, with the 5^th^ income quintile paying nearly three times as much total OPPs as those in the 1^st^ quintile. The AP for total OPPs decreases with income quintile, i.e. total payments for residential care represent a larger share of income for individuals in the lowest income quintile. The composition of total OPPs is differentiated across income quintiles. Individuals in the lowest quintile mostly pay asset-based OPPs while the two highest quintiles mostly pay income-based OPPs. Pflegeregress also represents the highest share of the annual mean income for those in the 1^st ^income quintile at nearly 107%. In comparison, Pflegeregress represents about 50% of the mean annual income in the highest quintile. Nonetheless, AP figures across quintiles are above the 25% threshold used by the WHO for catastrophic health spending [32].

**Table 1:** Distribution of OPPs in absolute value by income quintiles and home ownership status, 2015

Quintiles and types of OPPs	Mean absolute values (EUR)^a^, residential care users	AP (percentage), residential care users	Share of OPPs paid (percentage), residential care users
Income quintiles			
Total OPPs			
1^st^ quintile	13,325	156.1	18.0
2^nd^ quintile	20,379	145.7	16.9
3^rd^ quintile	24,329	138.9	19.2
4^th^ quintile	27,843	131.6	23.7
5^th^ quintile	37,430	113.4	22.3
Income-based OPPs			
1^st^ quintile	4,213	49.4	11.4
2^nd^ quintile	10,684	76.4	17.8
3^rd^ quintile	11,038	63.0	17.5
4^th^ quintile	16,441	77.7	28.1
5^th^ quintile	21,012	63.7	25.2
Asset-based OPPs (Pflegeregress)			
1^st^ quintile	9,112	106.8	24.4
2^nd^ quintile	9,695	69.3	16.0
3^rd^ quintile	13,291	75.9	20.8
4^th^ quintile	11,401	53.9	19.3
5^th^ quintile	16,418	49.7	19.5
Home-ownership			
Total OPPs			
Non-homeowner	17,350	96.7	34.2
Homeowner	41,408	206.7	65.8
Income-based OPPs			
Non-homeowner	10,661	59.4	52.8
Homeowner	11,787	58.8	47.2
Asset-based OPPs (Pflegeregress)			
Non-homeowner	6,688	37.3	21.8
Homeowner	29,621	147.9	78.2

Own calculations from the simulation model applied to 65 + Austrian sample of SHARE (2015). *N* = 2221. Ability to pay (AP) represents the average payment made as % of the group’s average income. Share of OPPs paid refers to the proportion of total revenue for that OPP type paid by the group. Notes:^a^Yearly values for 2015

Generally, figures in Table 1 show the 1^st^ and 2^nd^ quintiles contributing the lowest proportion of total- and income-based OPPs, while the 4^th^ and 5^th^ quintiles contribute the most. However, the 1^st^ income quintile contributes to approximately ¼ of all Pflegeregress paid, as it includes the largest proportion of care home residents, who on average spend the longest time in residential care. The substantial departure of wealth distribution from income can explain to a large extent why the Pflegeregress fell disproportionately on lower income individuals. There is only a moderate correlation between income and wealth among the older population in Austria (Spearman Rho = 0.425, *p* = 0.000), with many low-income individuals in a higher wealth quintile (see Appendix 3). Given the average assets held by each quintile, the exemption thresholds for asset-based OPPs did not sufficiently protect against asset deplection: the 1st income quintile on average held assets with a value of almost 5 times that of the highest possible exemption threshold.

In absolute terms, homeowners pay on average over twice as much for total OPPs and over four times as much for asset-based OPPs compared to non-homeowners. Conversely, income-based OPPs are relatively similar across these two groups. Homeowners contribute the largest proportion of total OPPs paid, driven entirely by the Pflegeregress, for which they contribute nearly 3/4 of total Pflegeregress payments.

We carried out an analysis of the intra-quintile distribution of asset-based OPPs paid, conditional on requiring residential care, to assess how OPPs are distributed within quintiles and in particular how concentrated payments are in just a few individuals within quintiles.^1^

**Fig. 1:**
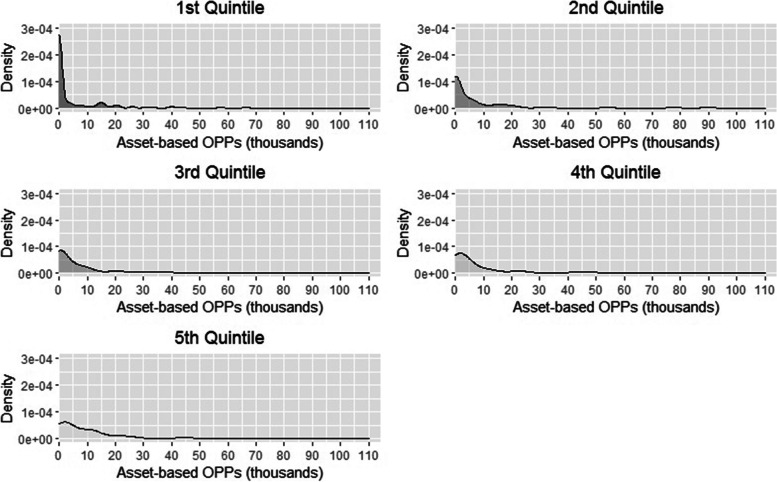
Intra-quintile distribution of asset-based OPPs for individuals requiring residential care. Each graph represents the density curve of asset-based OPPs (Pflegeregress) paid by residential care users in each quintile. As the probability of using residential care varies by quintile (i.e. those in the 1^st^ quintile are more likely to use residential care compared to the 5^th^ quintile), the number of individuals represented in each graph varies

The density curves for asset-based OPPs confirm that across all quintiles, most individuals who need residential care pay relatively little (i.e. under €10,000), while relatively few pay very high amounts (Fig. 1). Also noteworthy is that asset-based OPPs paid across quintiles are base heavy, especially for the 1^st^ quintile, albeit for different reasons: for the lower quintiles many individuals make limited or no payments at all as their assets are below the minimum threshold to pay the Pflegeregress; while for the upper quintiles many individuals have sufficiently high income to pay OPPs without spending down their assets. Despite this, a comparable number of individuals in the 1^st^ and 2^nd^ quintiles pay just as much (if not more in some cases) as those in the higher quintiles. The intra-quintile distribution of asset-based OPPs confirms that although fewer individuals are liable to pay these OPPs, the amounts payed are substantial.

**Fig. 2:**
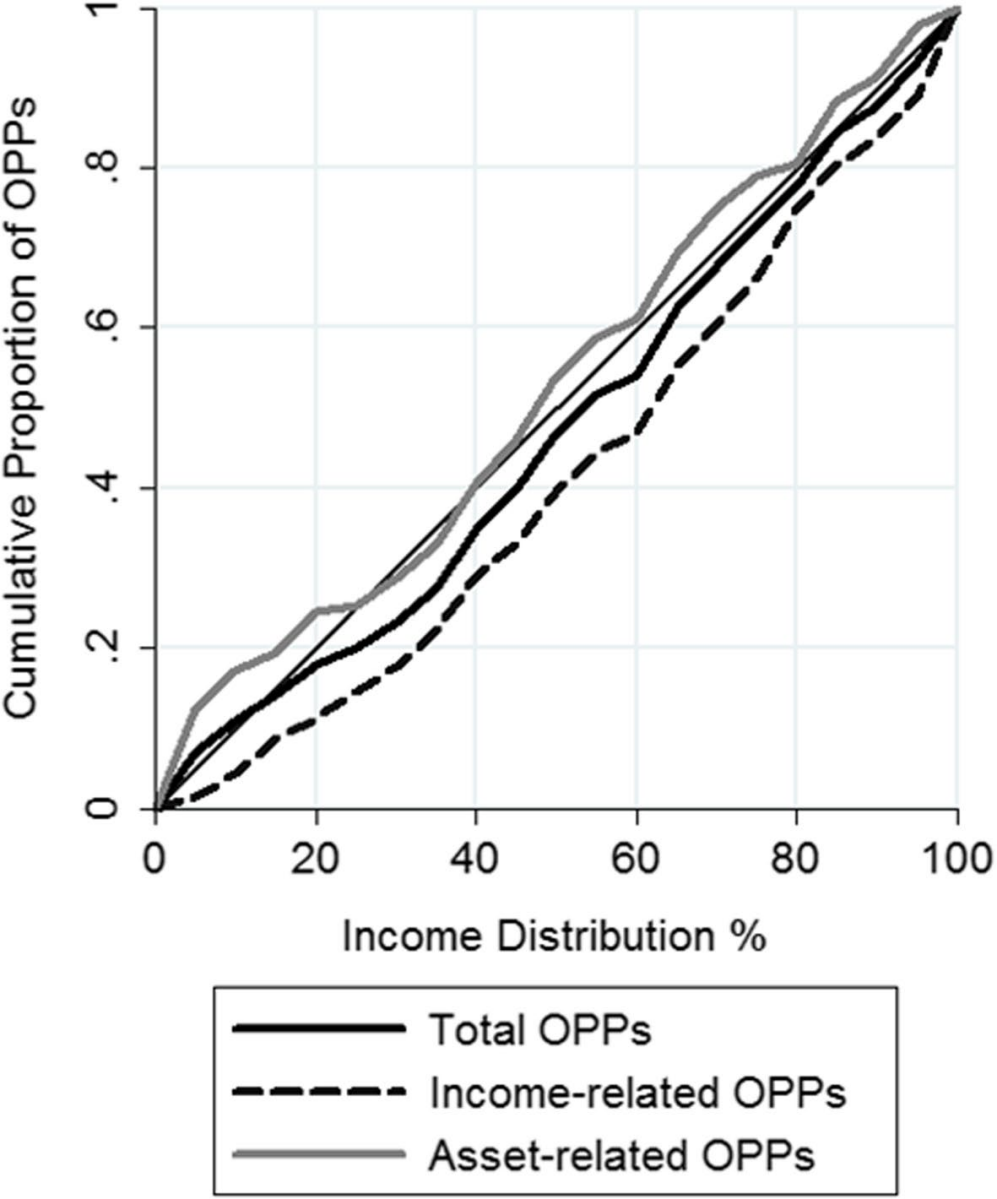
Concentration curves for different types of OPPs. The population is composed of the SHARE wave 6 sample aged 65 + used in the micro-simulation for 2015 (*N*  =  2221). Cumulative proportion of yearly OPPs paid (2015 values). Individuals are ranked according to their equivalized net income in 2015

The CI for total OPPs is positive, while the CI for the Pflegeregress is negative (i.e. payments are concentrated on the less affluent), albeit neither are statistically significant (first column, Table 2). The CI for income-based OPPs is markedly pro-rich and statistically significant (i.e. payments are concentrated on richer individuals). The CC for income-related OPPs is situated further below the 45-degree than the CC for total OPPs (Fig. 2). Conversely, the CC for asset-based OPPs lies mostly above the 45-degree line, save for between the 25^th^ and 40^th^ percentile of the income distribution, which crosses below the 45-degree line.

**Table 2:** Concentration indices for actual OPPs, needs-adjusted OPPs and inequity indices for the baseline scenario

	CI (Actual OPP)	CI (Needs adjusted OPP)	HI
Total OPP	0.057	-0.115***	0.172***
Income-related OPP	0.168**	-0.134***	0.301***
Asset-related OPP	-0.052	-0.097***	0.045

^*^p < 0.05; **p < 0.01;*** *p* < 0.001. *N* = 2221. *CI* Concentration Indices, *HI* Horizontal Indices

For all types of OPPs, needs-adjusted OPPs are markedly pro-poor reflecting the fact that use of residential facilities is concentrated among less affluent individuals (second column, Table 2). The HI for total and income-based OPPs after standardaising for need is positive and statistically significant, denoting the higher ability of higher income individuals to pay for residential care mostly through their income. Asset-based OPPs fall short of representing the actual concentration of use of residential facilities among poorer individuals, resulting in a positive (although not statistically significant) HI.

The alternative scenarios all leave the income-based OPPs unchanged, as each replaces only the asset-based OPPs of the baseline scenario by an alternative funding source for residential care. In contrast to the baseline scenario where only residential care users pay OPPs, both alternative scenarios decouples payments from use and broadens the financing base for residential care. Considering that costs are redistributed to include non-residential care users as well, the values for the AP by each quintile are significantly reduced for residential care users (Table 3, first column). Of the two alternative scenarios, contributions from residential care users would be the highest in proportion of their income under the inheritance tax scenario, especially among those in the middle to upper quintiles (between 3.3% and 8.1%). Residential care-users, regardless of quintile, would pay neglible proportions under the social insurance scenario (1.6%-1.7%). The APs by each quintile averaged across the entire population (both care-users and non-care users) are also neglible, reflecting the wider financing base of these scenarios (Table 3, second column).

**Table 3:** Distribution of OPPs for the alternative scenarios by income quintiles and home ownership status, 2015

Quintiles alternative scenarios	AP (%), residential care users	AP (%), all individuals 65 +	Share of OPPs paid (replacement of asset-based OPPs), all individuals 65 + _
Component replacing asset-based OPPs			
Inheritance tax scenario			
1^st^ quintile	0.1	1.5	2.9
2^nd^ quintile	< 0.1	0.6	2.1
3^rd^ quintile	8.1	4.6	39.5
4^th^ quintile	4.4	1.5	23.5
5^th^ quintile	3.3	1.4	32.0
Social insurance scenario			
1^st^ quintile	1.6	1.8	9.0
2^nd^ quintile	1.6	1.8	14.8
3^rd^ quintile	1.7	1.8	18.6
4^th^ quintile	1.6	1.8	22.4
5^th^ quintile	1.6	1.8	35.2
Home-ownership			
Inheritance tax scenario			
Non-homeowner	1.9	1.2	31.4
Homeowner	6.0	2.8	68.6
Social insurance scenario			
Non-homeowner	1.6	1.8	53.1
Homeowner	1.7	1.8	46.9

Own calculations from the simulation model applied to 65 + Austrian sample of SHARE (2015). *N* = 2221

In each of the alternative scenarios, the 1st and 2nd quintiles contribute the lowest proportion to the total revenue generated and substantially less than under the Pflegeregress (Table 4). They are particularly better off under the inheritance tax scenario, as most do not hold assets in excess of €300,000. On average the other quintiles would be worse off in any of the alternative scenarios, as they would pay a higher proportion of the total revenue generated than under the baseline scenario, except for the social insurance scenario for the 3^rd^ quintile.

**Table 4:** Concentration indices for actual OPPs, needs-adjusted OPPs and inequity indices for baseline and alternative scenarios

	CI (Actual OPP)	CI (Needs adjusted OPP)	HI
Baseline scenario	-0.052	-0.097***	0.045
Inheritance tax scenario	0.311***	-0.057**	0.369***
Social insurance scenario	0.260***	0.025***	0.235***

Note: *p < 0.05; **p < 0.01;*** *p* < 0.001. N = 2221. *CI* Concentration Indices, *HI* Horizontal Indices. Baseline scenario represents the asset-based OPPs prior to abolishment of the Pflegeregress

Both non-homeowners and homeowners who use residential care would be better off under each of the alternative scenarios, again due to the wider financing base of each of these scenarios. Homeowners in general would still contribute more than double that of non-homeowners of total payments in the inheritance tax scenario, albeit this share would be less than under the Pflegeregress. With a social insurance scheme in place, both non-homeowners and homeowners would contribute approximately the same proportion to total contributions paid as their respective share in the population.

Contrary to the negative CI for the Pflegeregress, the CIs for each alternative scenario are positive and statistically significant, meaning that each alternative scenario is progressive with payments concentrated on richer individuals (Table 4). This is also visible in the CC (Fig. A1 in Appendix 4) as both CCs are situated below the 45 degree line. The CI for the needs-adjusted OPPs in the social insurance tax scenario deviate from the baseline scenario in that they are pro-rich. In contrast, the CI for needs-adjusted payments in the inheritance tax scenario is statistically significant and negative, although lower in absolute value than the baseline CI. While both alternative scenarios decouple payments from needs and actual use of residential care, the inheritance tax more closely follows the needs distribution, which is concentrated on less affluent individuals than the other alternative scenarios. Finally, the HIs for both alternative scenarios are positive and significant, indicating that richer individuals pay a majority of overall payments even after adjusting for need.

## Discussion

Asset-based OPPs partially rest on fairness arguments, as contributions to care costs should reflect individuals’ ability to pay and older people have significant accumulated assets that could be used to smooth consumption along the life-cycle and pay for care [33]. Individuals all across the income distribution in Austria were required to pay out of assets for residential care, however the Pflegeregress disproportionately fell on low-income individuals and was largely financed through housing assets. Many individuals in the 1^st^ and 2^nd^ quintile held assets in excess of the asset threshold and were therefore liable to pay substantial Pflegeregress payments. This is in contrast with most higher-income individuals, many holding assets themselves, who could cover residential care fees mostly from income alone. These findings closely resemble a previous U.S.-based study that simulated the distribution of Medicaid benefits and individuals required to spend their assets down to $2,000 before being entitled to benefits [15]. Medicaid is the needs-based U.S. benefit that covers residential care costs (board and lodging and assistance with ADLs) and is avaliable for those whose income and assets fall below certain thresholds (i.e. it is income and assets-tested). In that study, the authors found that increasing the asset threshold for eligibility to Medicaid would benefit less wealthy individuals by reducing the rate of spending-down among this group. Still, the intra-quintile analysis of asset-based OPPs showed that most individuals requiring residential care can expect to pay modest OPPs, while relatively few will pay inordinate amounts. Albeit a minority, even some individuals in the 5^th^ income quintile, would be liable to pay Pflegeregress, confirming the high costs associated with residential care.

The regressive nature of the Pflegeregress thus went against the universal principles (no means-testing) underpinning financing of care for older people in Austria. It stands then, that low-income individuals were, in relative terms, the largest beneficiaries of its abolishment. In constrast, a study that simulated a complete disregard of assets in England found beneficiaries to be more evenly distributed along the income distribution [12]. However, the threshold for asset-based OPPs in Austria was markedly lower (€4,000–12,000, while the equivalent in England is €32,000) and the correlation between the income and wealth distribution among older people weaker [1].

We also simulated the ‘road not taken’ in terms of alternative financing arrangements that would have made up for the revenue shortfall caused by the abolishment of the Pflegeregress. As mentioned before, these alternative scenarios were based on other empirical studies and the discussion leading up to the abolishment of the Pflegeregress [10, 11, 24]. These scenarios decouple payment from actual use and not only would they reduce the uncertainty that is associated with asset-based OPPs (or any OPP for LTC for that matter) [12, 34], but our results show that each of the alternatives is significantly more progressive than the baseline scenario. If implemented, each would have entailed a re-distribution from healthy (i.e. those potentially not needing residential care) to less healthy older people, and through this a redistribution from higher to lower income quintiles in comparison with asset-based OPPs. The inheritance tax scenario however, would also disproportionately fall on the middle class, with the 3rd quintile covering the highest portion of payments than any quintile. Like in other studies [10], completely disregarding assets would leave homeowners better off under each of the alternatives, including the inheritance tax scenario.

This study also shows the potential of matching administrative and survey data and using micro-simulation to overcome data limitations and assess policy changes in public care systems. There are however, some caveats that should be considered. The analysis is based on a matched administrative and survey dataset and not on actual data on payers of Pflegeregress. The sensitivity analyses carried out suggest, however, that the simulation is close to the profile of actual users of residential facilities. Our simulations report annual payments and their distribution for a one year time horizon (2015). Other studies used stylized households or lifelong costs [2, 10, 12], which limit comparability of findings. Our findings do not consider behavioural changes brought by the abolishment of the Pflegeregress: residents of institutional care increased by 15% in 2018, the year asset-based OPPs were abolished [35, 36].

Our simulation shows the relevance of how financing of residential facilities is configurated from a distributional viewpoint, particularly for countries that impose asset-testing. Unless asset exemptions are set high enough [10, 15], asset-based OPPs are likely to disproportionately fall on least affluent individuals, for whom affordability of residential care will hinge on asset depletion [2, 13]. Given the findings of this study, one could conclude that setting asset-based OPPs for residential care in particular to ‘zero’ would be an optimal policy, given the regressive nature of such OPPs. As demonstrated by the healthcare literature on OPPs, however, zero OPPs are seldom an optimal level for cost-sharing, particularly if the price elasticity for residential care is not zero, due to moral hazard considerations [37]. Establishing the price elasticity of residential care in Austria was beyond the scope of our analysis, but the limited evidence that exists seems to point to a low price sensitivity for long-term care (LTC) [3, 5]. If this is confirmed, the main function of asset-based OPPs would be to act as a redistribution mechanim. As demonstrated above, the distribution of *Pflegeregress* was heavily influenced by residential care use and duration and as such, its role as a re-distributive policy is limited. Policy alternatives could include decoupling financing from use (e.g. earmarked inheritance taxes, or social insurance), which could compensate for lost revenue without the adverse distributional effects of Pflegeregress [11]. Moreover, as each of such alternatives would keep the current income-based OPPs in place, they would still be compatible with limiting moral hazzard in the use of residential care. The concentration of very high asset-based OPPs in just a few individuals, revealed by the intra-quintile distribution analysis, actually speaks of the feasibility of social insurance schemes to cover the risk of catasthropic expenditure with residential care. As mentioned above, we did observe a shift in demand for residential care facilities in Austria following the abolishment of the *Pflegeregress*, which our findings did not incorporate. There is ample evidence that sensitivity to changes in costs born by users – and the abolishment of the *Pflegeregress* would be one such example – varies across income levels. Conventional economic reasoning would state that reductions in care costs should have a larger impact on those individuals for which these represent a higher share of their income or consumption [37], but empirical studies offer contradictory evidence [38]. One cannot rule out that the shift in demand might have been concentrated on more affluent individuals, for whom informal care and/or home care use are now comparatively more expensive. This too would have possible distributional implications and would merit further research.

## Conclusion

Data unavailability have thus far limited the possibility to analyze the distributional impact of OPPs among residential care users. We use a novel method in this study that provides an alternative approach to overcome these data limitations. The findings of this study highlight the need to consider the distribution of costs with residential care facilities, particularly OPPs, in the debate on how best to finance costs associated with ageing. Unless asset-exemption thresholds are set high enough, asset-based OPPs for residential care, which are currently widespread across Europe, risk disproportionately impacting lower income individuals who have even modest levels of savings and may therefore be an inefficient way to target payments to more affluent individuals. Policy alternatives, such as an earmarked inheritance tax or social insurance scheme, could compensate for lost revenue without the adverse distributional effects of Pflegeregress by redistributing payments and decoupling use from costs.

## Supplementary Information

**Additional file 1. **

## Abbreviations

ADL: Activities of Daily Living
CC: Concentration Curves
CI: Concentration Indices
IADLs: Instrumental Activities of Daily Living
LTC: Long-term care
OPPs: Out-of- pocket payments

## Footnotes

1 The density curves represent payments conditional on requiring residential care and as such the number of observations in each quintile varies according to the probability of using residential care (i.e. there are more individuals represented in the density curve for the 1^st^ quintile as these are disproportionately represented among residential care users).
