# Supplementary material for: From each according to means, to each according to needs? Distributional effects of abolishing asset-based payments for residential care in Austria
Source: Int J Equity Health. 2022 Mar 19;21:39. doi: 10.1186/s12939-022-01639-y (PMC8934458; doi:10.1186/s12939-022-01639-y)
Supplement: Supplementary file 1 — Additional file 1. [file 12939_2022_1639_MOESM1_ESM.docx]

**APPENDIX I: Additional details on the data matching and micro-simulation model**

*Matching administrative data to SHARE data*

In a first step, the remaining life expectancy spent in a LTC facility (i.e. duration of residential care use), stratified by age, gender and care level was estimated using life tables from Statistik Austria^13^ with information on the population $Px_{i}$ at the beginning of 2015 and the number of deaths $Dx_{i}$ in that year. The Sullivan Method was adapted to obtain both total life expectancy and life expectancy spent in residential care for each age.^15^

The proportion of those in the age group $\pi_{i}$ living in a residential facility, stratified by age and care level, was taken from the data on care allowance recipients^12^ and from the data on the probability of being in a residential facility.^14^ The person years lived without being in a residential facility ${\sigma x}_{i}$ at age$x_{i}$ was then calculated by subtracting the proportion of those in a residential facility $\pi_{i}$ from 1 and multiplying it by the person years lived ${Lx}_{i}$ that was previously calculated: ${\sigma x}_{i}=\left( 1-\pi_{i} \right)*{Lx}_{i}$. The total years lived without being in a residential facility ${\theta x}_{i}$ from age$x_{i}$ was then calculated by summing person years lived without being in a residential facility ${\sigma x}_{i}$ from$x_{i}$ onward:

$${\theta x}_{i}= \sum_{x_{i}}^{99+} {\sigma x}_{i}$$

Residential care-free life expectancy${\beta x}_{i}$ equals the total years lived without being in a residential facility ${\theta x}_{i}$ divided by the probability of survival to that age ${lx}_{i}$. Finally, the total expected time spent in a residential facility ${\Delta x}_{i}$ in years was calculated by subtracting the residential care-free life expectancy ${\beta x}_{i}$ from the total life expectancy ${ex}_{i}$ calculated before.

In the second step, the expected time spent in residential care given a particular age, gender and care level, was matched with individuals aged 65+ in the Survey of Health, Ageing and Retirement in Europe (SHARE, wave 6) for Austria (N=2221) (Table A1). To this end, we used the process detailed by Brugiavini et al.^17^ to assign likely care levels of the Austrian care allowance to each individual in SHARE, based on the needs profile of each individual. First, time allotments (i.e. ‘needs’ as defined by the Austrian care allowance) were allocated for each task to individuals in SHARE that report difficulties with said task. While not all tasks listed by the legislation could be accounted for in SHARE (i.e. colostomy care, catheter care, enemas, etc.), a large majority could (Table A2). Mental health/disorders were also considered, with individuals with Alzheimer diagnoses or behaviour disorders being attributed the corresponding number of hours per month as designated by the legislation.

Table A1: Descriptive statistics for SHARE sample used in the simulation

| Variable | Percentage (mean; SD) |
| --- | --- |
| Age | (74.6; 7.0) |
| Gender: |  |
| Female | 57.5 |
| Male | 42.5 |
| Education: |  |
| Primary education | 15.5 |
| Secondary education | 59.8 |
| Tertiary education | 24.8 |
| Health variables: |  |
| No ADLs | 85.1 |
| ADLs>=1 | 14.9 |
| No IADLs | 72.9 |
| IADLS>=1 | 27.2 |
| Good or better self-reported heatlh | 60.9 |
| Less than good self-reported health | 39.1 |
| Homeownership |  |
| Homeowner | 56.3 |
| Non-homeowner | 43.7 |
| Equivalised household net income | (21,954.1; 13556.47) |
| Net worth | (146223; 253956.1) |
| No. of observations | 2221 |

Unweighted results. Individuals aged 65 or older.

Table A2: Care tasks used to assign time allotments to observatios in SHARE

| Care Task | Time assigned (hours) | Question in SHARE |
| --- | --- | --- |
|  |  | Please tell me if you have any difficulties with these activities because of a physical, mental, emotional or mental problem. Exclude any difficulties you expect to last less than three months. |
| Daily body care | 25 | Bathing or showering |
| Preparation of meals | 30 | Preparing a hot meal |
| Taking meals | 30 | Eating, such as cutting up your food |
| Defecation | 30 | Using the toilet, including getting up or down |
| Dressing & Undressing | 20 | Dressing, including putting on shoes and socks |
| Cleaning for incontinence | 20 | Use of continence pads |
| Taking medication | 3 | Taking medications |
| Mobility aid in the narrow sense | 15 | Walking across a room |
| Shopping for food and medicines | 10 | Shopping for groceries |
| Cleaning home and personal effects | 10 | Doing work around the house or garden |
| Heating the living space and procurement of fuel | 10 | Doing work around the house or garden |
| Care of underwear and towels | 10 | Doing work around the house or garden |
| Mobility aid in broader sense | 10 | Using map to figure out how to get around in a strange place |
| Cognitive impairment | 25 | Has a doctor ever told you had/do you currently have:  Alzheimer's disease, dementia, organic brain syndrome, senility or any other serious memory impairment OR other affective or emotional disorders, including anxiety, nervous or psychiatric problems |
| Motivational talks | 10 | EURO-D scale |

Note: The following tasks (time allotment in hours) have no equivalent variables in SHARE and were not used: colostomy care (7.5), cannula tube care (5), catheter care (5), enemas (15), emptying and cleaning toilet chair (10).

Time allotments were summed up for each individual and the care allowance eligibility thresholds were then applied to assign each individual to a hypothetical care level (i.e. Pflegestufe) according to the number of hours of care ‘needed’ (Table A3). Care levels 5-7 are all characterized by requiring at least 180 hours of care per month, therefore other factors in addition were considered to distinguish between these higher care levels, including Activities of Daily Living (ADLS), Instrumental Activities of Daily Living (IADLS), mobility issues and number of chronic conditions.

Table A3: Eligibility criteria for the care allowance, 2015

| Care Level | Care requirement |
| --- | --- |
| 1 | More than 65 hours/month |
| 2 | More than 95 hours/month |
| 3 | More than 120 hours/month |
| 4 | More than 160 hours/month |
| 5 | More than 180 hours/month |
| 6 | More than 180 hours/month and care measures cannot be coordinated in terms of time and permanent presence of a caregiver during the day and night is needed |
| 7 | More than 65 hours/month and practical inability to move 4 extremities |

Source: Bundespflegegeldgesetz § 4 (2015), Austria

At this stage, all individuals in the SHARE dataset have been assigned a hypothetical care level given their reported difficulties with care tasks, but this does not account for possible non-take up or unmet needs arising from deviations between self-reported need (i.e. in SHARE) and assessed need. Therefore, the actual distribution of beneficiaries of the care allowance (and consequently, also the distribution of those in residential care – both distributions derived from Pflegedienstleistungsstatistik data) were superimposed onto the SHARE dataset to ensure the SHARE distribution of care levels and residential care users mirrors that of the real-life distribution. For example, if 5% of individuals between 70-74 received the highest level of care allowance in Austria, this care level is assigned to 5% of individuals of the same age cohort in the SHARE sample. To perform a match between the real-life distribution of care beneficiaries and the care levels we assigned in SHARE taking care level, gender and age category as the distance-minimizing variables, the R package ‘NND.hotdeck’ was employed. We used the Manhattan distance function to minimize the distance between variables, due to the categorical and discrete nature of the variables used in the match. Given the representative nature of the SHARE sample, matching the distributions results in a near perfect match on age and gender. Expected time spent in a residential facility (previously estimated using the Sullivan method) in 2015 was then assigned to each individual in SHARE according to gender, age and newly matched care level (see Table A4).

Table A4: Average expected duration of residential care (months) per quintile, gender and care allowance level

| Care level | | Income quintile | | Gender | |
| --- | --- | --- | --- | --- | --- |
| 1 | 0.97 | 1^st^ quintile | 7.45 | Women | 7.35 |
| 2 | 1.37 | 2^nd^ quintile | 7.19 | Men | 5.84 |
| 3 | 3.17 | 3^rd^ quintile | 6.19 |  |  |
| 4 | 12.65 | 4^th^ quintile | 7.12 |  |  |
| 5 | 13.59 | 5^th^ quintile | 6.06 |  |  |
| 6 | 12.23 |  |  |  |  |
| 7 | 8.26 |  |  |  |  |

Source: Own calculations from the simulation model applied to 65+ Austrian sample of SHARE (2015). N=2221.

*Parameters of the micro-simulation model*

To estimate OPPs, the micro-simulation model uses the 2015 rules of Pflegeregress to simulate income liable to be used in determining OPPs (e.g. considering the living allowance exception for 2015) and the amount of asset exemptions by region (Table A5). Using the tariffs set by regional governments, annual costs with residential care in 2015 range between 37,194 EUR and 72,866 EUR. These must be covered from residents’ income and Pflegegeld, except for a monthly living allowance exception (i.e. income-based OPPs), as well as assets (i.e. asset-based OPPs), with the remainder being funded by social assistance. The pocket allowance for 2015 allowed care users to keep a fixed amount of 45.18 euros per month (10% of the level 3 of the care allowance), their 13^th^ and 14^th^ annual payment (old-age pensions in Austria are paid 14 times a year) and 20% of their remaining pension income. Pocket allowance was set at a minimum of 214.07 euros and a maximum of 481.78. By applying these rules to our assembled dataset, we can simulate OPPs paid and the effect of asset-based OPPs’ abolishment.

Table A5: Exempted asset threshold for Pflegeregress and average cost of residential care by Austrian regions, 2015

| Region | Exempted asset threshold (Euros) | Average annual cost of residential care (Euros) |
| --- | --- | --- |
| Vienna | 4,000 | 72,866 |
| Burgenland | 8,450 | 50,880 |
| Carinthia | 4,222 | 50,880 |
| Lower Austria | 12,667 | 48,228 |
| Upper Austria | 7,300 | 45,234 |
| Salzburg | 5,235 | 50,880 |
| Styria | 7,000 | 37,193 |
| Tyrol | 7,000 | 37,193 |
| Vorarlberg | 10,000 | 50,880 |

Source: BMASK (2017) Österreichischer Pflegevorsorgebericht 2016. Wien: BMASK.

The micro-simulation model relies on the following assumptions:

- If an individual enters residential care in 2015, they spend their entire assigned duration in residential care all at once. Time spent in residential care is not divided over the remaining course of their lifetime.
- All household wealth is used in calculating liable asset-related OPPs (i.e. including housing assets, regardless of whether the individual lives with a spouse that continues to live in the house).

*Simulating the alternative financing scenarios*

Both alternative financing scenarios were calculated endogenously according to income and wealth held by Austrians aged 65+ in SHARE. For the social insurance contribution scenario, an additional social insurance contribution of 2.27% is simulated for those aged 65+. It mirrors the design of other social insurance contributions in Austria and is applied to the sum of all income sources (i.e. pension income plus income from employment and self-employment) exceeding €405,98 per month up to a maximum contribution base of €4,650 per month. The inheritance tax scenario is simulated as a tax rate of 31.48%, applied to the net wealth ((i.e. including housing assets net of debts and other financial assets) above the threshold of €300,000 that is held by individuals that are expected to die in the given year. The probability of dying was calculated using information from the periodic life table for 2015 (Statistik Austria 2017a), stratified by gender, age and care level.

**APPENDIX II: Sensitivity analysis**

There are no publicly available data on actual payers of asset-based OPPs in Austria with which the distribution of simulated OPPs from the micro-simulation model could be compared. However, it was possible to compare the income distribution of the sample used in the micro-simulation with data on actual users of residential care provided by the Fonds Soziales Wien (FSW) (for further information on the latter see ^11^). Data from the FSW includes all persons in the region of Vienna who used residential care in 2011 with information on their income. This income distribution was updated for 2015 and compared with the one for the whole of Austria resulting from the matching method described in Annex I. Results are shown in Table A5. Sample size precluded the comparison of the FSW data with the SHARE sample for Vienna only. We conclude that the matching method used on administrative and SHARE data that underpins the micro-simulation yields a similar profile of simulated users of residential care whose income distribution is comparable to actual figures from Vienna.

Table A6.: Deviation (in percentage) between the income distribution of users of residential care in FSW data and our simulation sample

| Percentile of income | 10% | 25% | mean | 50% | 75% | 90% | 95% |
| --- | --- | --- | --- | --- | --- | --- | --- |
| Average deviation from FSW data | --† | -3.72 | 0.13 | -2.85 | -8.50 | -9.98 | -0.47 |

Source: Own calculations using data from FSW, Statistics Austria and SHARE.
Notes: † No income-related information for the 1^st^ decile.
Negative values mean that income in the simulation sample was lower than in the FSW data for users of residential care and vice-versa for positive values.

**APPENDIX III: Distribution of income and wealth among Austrians 65+ (SHARE)**

Table A6: Distribution of wealth and income in Austria

|  |  | Wealth | | | | | Average assets per household (Euros) |
| --- | --- | --- | --- | --- | --- | --- | --- |
|  |  | 1st quintile | 2nd quintile | 3rd quintile | 4th quintile | 5th quintile |  |
| Income | 1st quintile | 39.6 | 22.1 | 15.1 | 17.3 | 5.7 | 67,678 |
|  | 2nd quintile | 26.5 | 23.3 | 19.6 | 20.1 | 10.4 | 98,530 |
|  | 3rd quintile | 18.2 | 25.7 | 20.9 | 19.5 | 15.5 | 136,228 |
|  | 4th quintile | 11.6 | 19.0 | 22.2 | 21.41 | 25.7 | 211,220 |
|  | 5th quintile | 4.0 | 10.1 | 21.8 | 21.3 | 42.5 | 47,429 |
| Spearman Rho: r= 0.425 (p-value=0.000) | | | | | | |  |

Source: Own calculations based on 65+ Austrian sample of SHARE (2015). N=2221. Weighted results.

**APPENDIX IV: Concentration curves for baseline and alternative scenarios**

Figure A1: Concentration curves for the Pflegeregress (baseline) and alternative scenarios.

Notes: N=2221. Cumulative proportion of OPPs paid. Ranking variable is equivalized net income.
